# Diagnosis of Epstein-Barr and cytomegalovirus infections using decision trees: an effective way to avoid antibiotic overuse in paediatric tonsillopharyngitis

**DOI:** 10.1186/s12887-023-04103-0

**Published:** 2023-06-17

**Authors:** Andrea Tímea Takács, Mátyás Bukva, Csaba Bereczki, Katalin Burián, Gabriella Terhes

**Affiliations:** 1 Department of Pediatrics and Pediatric Health Center, University of Szeged, Korányi fasor 14-15, Szeged, 6725 Hungary; 2 Data Science and Me Ltd, Kecskemét, Hungary; 3 Institute of Clinical Microbiology, University of Szeged, Szeged, Hungary

**Keywords:** Tonsillopharyngitis, Antibiotic treatment, Infectious mononucleosis, Epstein–Barr virus (EBV), Cytomegalovirus (CMV), Elevated transaminases, Decision tree, Machine learning

## Abstract

**Background:**

The incidence of tonsillopharyngitis is especially prevalent in children. Despite the fact that viruses cause the majority of infections, antibiotics are frequently used as a treatment, contrary to international guidelines. This is not only an inappropriate method of treatment for viral infections, but it also significantly contributes to the emergence of antibiotic-resistant strains. In this study, EBV and CMV-related tonsillopharyngitis were distinguished from other pathogens by using machine learning techniques to construct a classification tree based on clinical characteristics.

**Materials and methods:**

In 2016 and 2017, we assessed information regarding 242 children with tonsillopharyngitis. Patients were categorized according to whether acute cytomegalovirus or Epstein-Barr virus infections were confirmed (n = 91) or not (n = 151). Based on symptoms and blood test parameters, we constructed decision trees to discriminate the two groups. The classification efficiency of the model was characterized by its sensitivity, specificity, positive predictive value, and negative predictive value. Fisher’s exact and Welch’s tests were used to perform univariable statistical analyses.

**Results:**

The best decision tree distinguished EBV/CMV infection from non-EBV/CMV group with 83.33% positive predictive value, 88.90% sensitivity and 90.30% specificity. GPT (U/l) was found to be the most discriminatory variable (*p <* 0.0001). Using the model, unnecessary antibiotic treatment could be reduced by 66.66% (*p = 0.0002)*.

**Discussion:**

Our classification model can be used as a diagnostic decision support tool to distinguish EBC/CMV infection from non EBV/CMV tonsillopharyngitis, thereby significantly reducing the overuse of antibiotics. It is hoped that the model may become a tool worth considering in routine clinical practice and may be developed to differentiate between viral and bacterial infections.

**Supplementary Information:**

The online version contains supplementary material available at 10.1186/s12887-023-04103-0.

## Introduction

Acute pharyngitis is a frequent symptom of upper respiratory tract infections in both children and adults [1]. It is most commonly associated with viral infections such as adenoviruses, influenza and parainfluenza viruses, cytomegaloviruses (CMVs), and Epstein-Barr virus (EBV), but 15–30% of cases are also caused by bacteria such as group A streptococcus (GAS) [1, 2].

In clinical practice, despite the prevalence of viral infections, antibiotics are typically prescribed in 76% of cases, which is contrary to international guidelines and contributes significantly to the emergence of antibiotic-resistant strains [3]. Infectious mononucleosis (IM), which is caused by CMV and EBV, is characterized by this antibiotic overuse. Although IM has some distinctive symptoms (such as exudative pharyngitis, hepatosplenomegaly, and lymphadenopathy), other pathogens, including bacterial infections, are difficult to distinguish from IM. In practice, 53.10-72.69% of patients with IM receive antibiotics for a diagnosed bacterial infection [4, 5].

Given the foregoing, it is becoming increasingly important to differentiate between pharyngitis caused by viral and bacterial infection, or to use algorithms that can confidently identify specific viral/bacterial infections). Although the FeverPAIN and Centor scoring methods are widely utilized in clinical practice, their diagnostic accuracy is low due to overlapping symptoms if the patient’s score does not fall into a truly high-risk category [6].

In this study, we propose machine learning-based decision trees that can be used to distinguish EBV or CMV infections from patients in whom EBV or CMV is not confirmed, thereby reducing unnecessary antibioticuse.

## Materials and methods

### Patients

This study was conducted between January 2016 and December 2017. Patients with tonsillopharyngitis with EBV and CMV serologic evaluations (anti-VCA IgM, anti-VCA IgG, anti-EBNA IgG, anti-CMV-IgM, and CMV-IgG) (n = 242) were included. They were admitted to the Department of Paediatrics at the University of Szeged in Hungary for outpatient and inpatient care.

Data analysis included past medical history, clinical findings on presentation, indication for EBV and CMV serologies, findings of biochemical tests (full blood count, liver function tests and C-reactive protein) therapeutic interventions, and demographic data. The patients were all 0–18 years of age at first presentation.

We excluded patients from the study if no past medical history was available or if neither EBV nor CMV serologies were performed on presentation. We used the following case definition of upper respiratory tract infection: the admitted patient had fever and/or sore throat, cervical lymphadenopathy, and inflamed pharynx with or without exudate.

To perform the analyses, patients were divided into two groups (EBV/CMV and non-EBV/CMV) according to whether or not they had confirmed EBV or CMV.

### Microbiological investigations

EBV and CMV specific antibodies were detected by using an ETI-MAX 3000 microtiter plate analyzer (DiaSorin, Italy). Epstein-Barr Viral Capsid Antigen (VCA) Enzyme-linked immunosorbent assay (ELISA) immunoglobulin M (IgM) and G (IgG) (Vircell, Spain), and Epstein-Barr Nuclear Antigen (EBNA) ELISA IgG (Vircell, Spain) were used to detect EBV specific antibodies. Cytomegalovirus ELISA IgM capture and IgG (Vircell, Spain) were used to detect CMV-specific antibodies. Primary EBV infection was confirmed by anti-VCA IgM positive/borderline results, and anti-VCA IgG-positive result with negative anti-EBNA IgG test results. In case of isolated anti-VCA IgM results, control serology was recommended. The presence of anti-VCA IgG and anti-EBNA IgG with negative anti-VCA IgM serology indicated a past infection. Anti-CMV IgM was positive, and seroconversion was detected in the second specimen of the primary CMV infection. Past infection was confirmed by the presence of anti-CMV-specific IgG without anti-CMV IgM.

To exclude other causes, throat swabs were cultured using 5% sheep Columbia blood agar (bioMérieux, Marcy l’Etoile, France), chocolate (PolyViteX, bioMérieux, Marcy l’Etoile, France), and Sabouraud agar. Agar plates, except Sabouraud agar, were incubated at 37 °C in a 5% CO_2_ cabinet for 24 h. In the presence of *Streptococcus pyogenes*, identification of the isolated strain was performed by Matrix-Assisted Laser Desorption/Ionization Time-Of-Flight Mass Spectrometry (MALDI–TOF MS). Antibiotic susceptibility testing and evaluation of the results were performed according to the European Committee on Antimicrobial Susceptibility Testing (EUCAST) recommendations.

### Biochemical tests

We used Sysmex reagent to evaluate the full blood count on a Sysmex XE-2100 automated analyzer (Sysmex Europe GmbH, Germany). Alanine aminotransferase (GPT), aspartate aminotransferase (GOT) and C-reactive protein (CRP) analysis was performed using the standardized method of the International Federation of Clinical Chemistry (IFCC) on a ROCHE Modular P800 analyzer (Roche, Switzerland).

### Statistical analysis

To construct the classification trees, symptoms were used as categorical variables and laboratory results as continuous variables. 80% of the data served as a training data set, and the remaining 20% were used to test the constructed trees. During the construction of the trees, 5 cases had to be included in at least one node to prevent overfitting and to ensure the generalizability of the tree. The variables used for the trees were automatically selected by the algorithm based on information gain values.

Three classification trees were created: one based on symptoms, one based on blood test results, and one based on all of these variables. Age was entered into all models.

The intention behind creating a model based on symptoms is that the study aims to offer a model that can be applied quickly in a non-invasive way – even before blood test results are available – to support decision making about antibiotic treatment.

Classification efficiency was characterized by classification accuracy.

For univariable statistical analyses, we used Welch’s and Fisher’s exact test for continuous and discrete variables, respectively. We calculated 95% confidence intervals (CI) for the difference in means and for the odds ratio (OR). Receiver operating characteristic (ROC) analysis was performed on the GPT (U/l) variable and the area under the curve (AUC) and associated 95% confidence intervals were calculated. The optimal cut-off point was determined by the highest Youden index (sensitivity + specificity − 100).

Orange 3.32 and GraphPad Prism 9 were used to perform the statistical analyses, machine learning methods and create plots.

*p* < 0.05 was considered significant.

The investigation was approved by the local ethics committee (number of ethical permission: 124/2016 SZTE).

## Results

### More than a third of patients with confirmed EBV and/or CMV infection

EBV and/or CMV infection was confirmed in 37.60% of patients (91 of 242). These patients were assigned to the EBV/CMV group. Within the EBV/CMV group, serology confirmed acute EBV in 30.17% percent of patients (73 of 242) and primary CMV infections in 2.48% of patients (6 of 242). Of all patients, 4.96% (12 of 242) were found to be positive for anti-CMV IgM and anti-EBV VCA IgM, but negative for anti-CMV IgG, anti-VCA IgG, and anti-EBNA IgG. Repeated CMV and EBV serology were not performed; therefore, primary CMV or EBV infections were suspected based on clinical and initial laboratory findings.

In the EBV/CMV, 40.70% (37 of 91) of patients underwent bacteriological culture using a throat swab. Only 3 of the patients tested positive for *S. pyogenes*, *Streptococcus agalactiae* or *Candida albicans*.

Patients with no confirmed EBV and/or CMV infection were assigned to the non-EBV/CMV group (62.40%, 151 of 242). Of 151 patients, 60 patients had throat bacteriological cultures and only 5 patients tested positive for *S. pyogenes* and 1 patient for *C. albicans*. Adenoviral infection was confirmed in 5 patients, while influenza virus A and respiratory syncytial virus in 2 and 1 patient, respectively.

### Lymphadenopathy, hepatosplenomegaly exudation, sore throat are characteristic symptoms for EBV/CMV group

Examining the symptoms, prevalence of lymphadenopathy were found to be 27.02% higher (82.32% vs. 54.30%) in the EBV/CMV group, compared to the non-EBV/CMV group (OR = 3.66 [95% CI: 1.98 to 6.79], *p* < *0.0001).* Hepatosplenomegaly was also 26.24% more common in the EBV/CMV group (56.04% vs. 29.80%) than in the non-EBV/CMV group (OR = 3 [95% CI: 1.75 to 5.16], *p* < 0.0001). Exudation was identified 18.26% more often among the EBV/CMV patients (72.53% vs. 54.30%, OR = 2.22 [95% CI: 1.27 to 3.89], *p* = 0.006).

Cough (OR = 0.42 [95% CI: 0.22 to 0.81], *p* = 0.009) and nasal discharge (OR = 0.41 [95% CI: 0.22 to 0.77], *p* < 0.005) were found to be more characteristic for non-EBV/CMV group. For values for all symptoms, see the Additional file 1.

### Elevated levels of GOT and GPT were identified in the EBV/CMV group

**Fig. 1:**
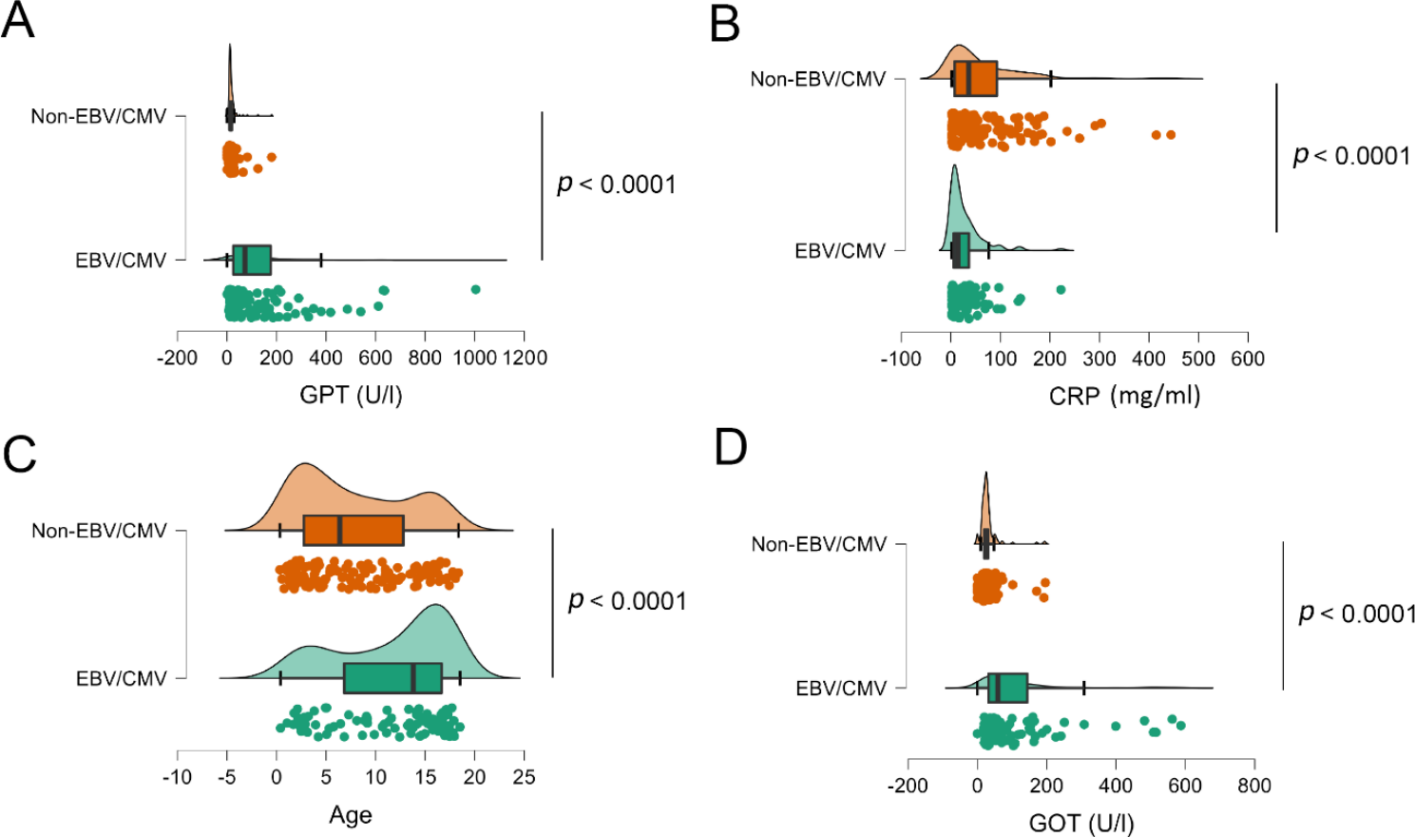
Raincloud plot for continuous variable. The raincloud plot shows the density and quartiles of the GPT (**A**), CRP (**B**), age (**C**), and GOT (U/I) variables. The different colours indicate the EBC/CMV (in green) and non-EBC/CMV (in orange) groups

Examining the blood test results and age, elevated GOT and GPT level, and highest age on average were identified among the EBV/CMV patients (Fig. 1). The EBV/CMV group had 8.05 times higher GPT levels than the non-EBV/CMV group (137.46 ± 172.12 U/l vs. 17.60 ± 19.11 U/l, *p* < 0.0001). Similarly, the average GOT level was found to be 3.54 times higher in EBV/CMV patients (106.56 ± 124.21 U/l vs. 30.07 ± 26.46 U/l, *p* < 0.0001), and they were 1.53 times older on average compared to non-EBV/CMV patients (11.80 ± 5.55 year vs. 7.71 ± 5.46 year, *p <* 0.0001).

The average CRP level was higher in the non-EBV/CMV group (27.88 ± 35.65 mg/l 63.42 ± 76.77 mg/l, *p* < 0.0001). No significant difference was found for WBC (for the descriptive statistics and detailed parameters see the Additional file 2).

### Blood test results are more suitable for identifying EBV/CMV infection than symptoms

To create the classification trees, data set was splitted into training and test sets, resulting in 80% of patients being assigned to the training set (193 of 242) and 20% to the test set ( 49 of 242 ).

In the train set, 62.70% of the patients were non-EBV/CMV (121 of 193), and 37.30% belonged to the EBV/CMV group (72 of 193). In the test set, 61.27% (31 of 49) and 36.73% (18 of 49) of the patients belonged to the non-EBV/CMV and EBV/CMV group, respectively.

After splitting the data, three classification trees were built based on symptoms, blood test results and all of the variables together. The trees were constructed using the train set, and the resulting models were tested on both train and test sets.

**Fig. 2:**
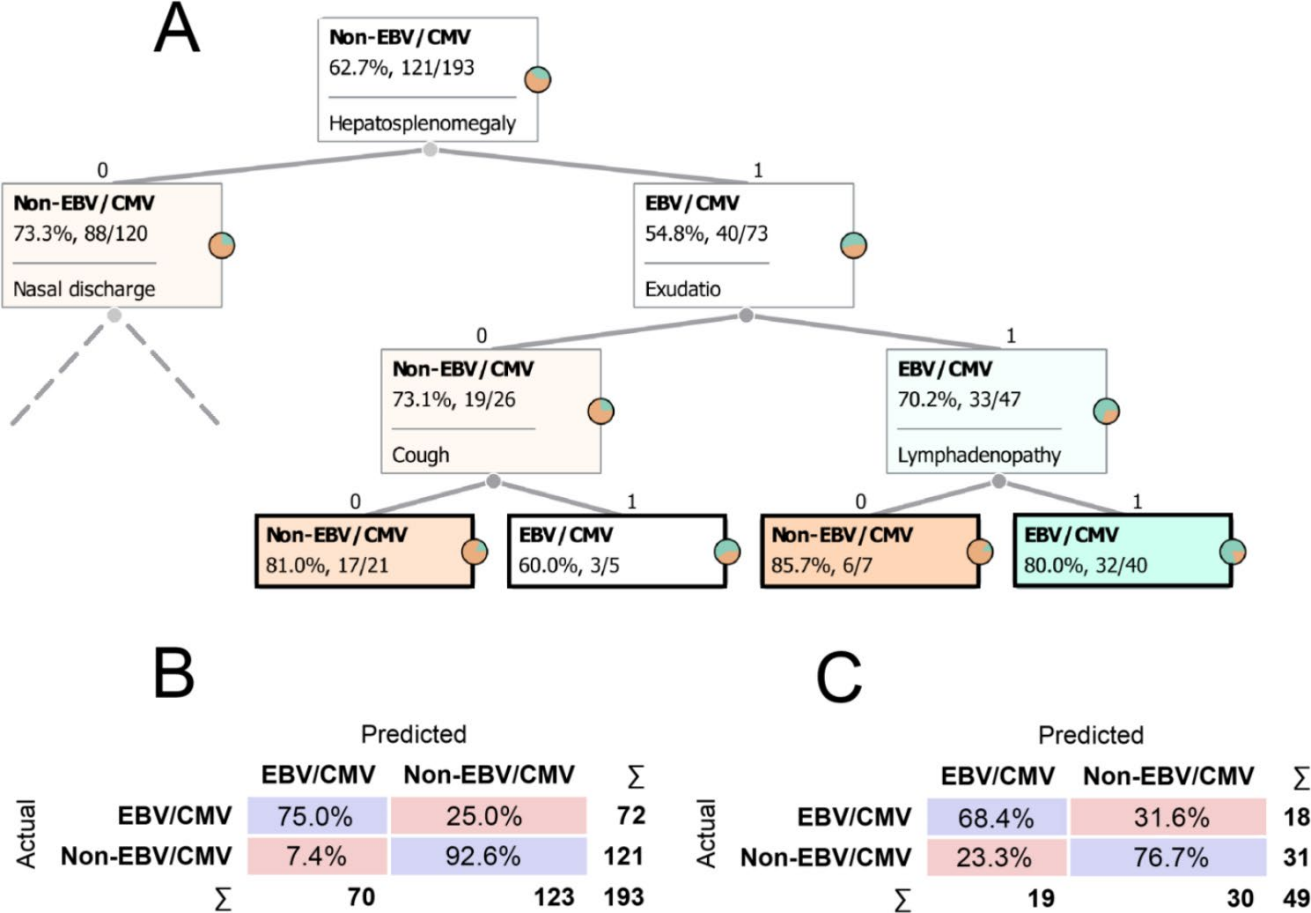
Classification tree based on symptoms. Part of the symptom-based classification tree (**A**): the nodes (rectangles) show the name and proportions of the most frequent group in each step. In the node at the top of the tree, the entire train set (n = 193) is still unpartitioned, in which non-EBV/CVM cases were the most common (62.70%, 121 of 192). At each step, patients are separated according to given symptoms to form more homogeneous nodes. A 0 denotes the absence of symptoms, while a 1 denotes their presence. Different intensities of the orange and green colours represent the percentage of non-EBV/CMV and EBV/CMV patients, respectively. Terminal nodes are marked with a black frames. The grey dashed line indicates that only part of the tree is shown. It is important to note that the classification is based on the whole tree. Confusion matrices for test (**B**) and train set (**C**): each row of the matrices represents the instances in an actual class while each column represents the instances in a predicted class. Diagonally, the percentage of the correct classification is shown in blue. The percentage of errors is indicated in red. For example, a total of 72 EBV/CMV patients are shown in panel B, and of these 72, 75% were classified by the model into the EBV/CMV group and 25% into the Non-EBV/CMV group

To build the symptom-based tree, the algorithm selected the soar throat/dysphagia, nasal discharge, exudation, cough, hepatosplenomegaly and lymphadenopathy. The best results were obtained with 4 levels (Fig. 2A; see the Additional file 3 for the full classification tree).

Running the model on the train dataset gave an average classification efficiency of 83.80% (Fig. 2B). The model classified 75.00% of EBV/CMV patients correctly and 25% incorrectly into the non-EBC/CMV group.

For the test set, the model correctly classified 68.40% of EBV/CMV patients, while it was wrong in 31.60%, resulting in an average classification accuracy of 72.55% (Fig. 2C). The positive and negative predictive values (the chances that a patient is EBV/CMV if he/she is classified as EBV/CMV by the model nad vice versa) calculated for the test data set was 63.16% and 80.00%, respectively.

The co-presence of hepatosplenomegaly, exudation, lymphadenopathy were found to be the most prominent features for the EBV/CMV group (Fig. 2A). This combination was observed in 40 patients in the train set, of which 80.00% belonged to the EBV/CMV group. In contrast, the absence of hepatosplenomegaly but the presence of nasal discharge and sore throat/dysphagia proved to be a particularly characteristic combination for the non-EBV/CMV group. This combination was observed in 34 patients, of which 94.10% (32 of 34) belonged to the non-EBV/CMV group.

**Fig. 3:**
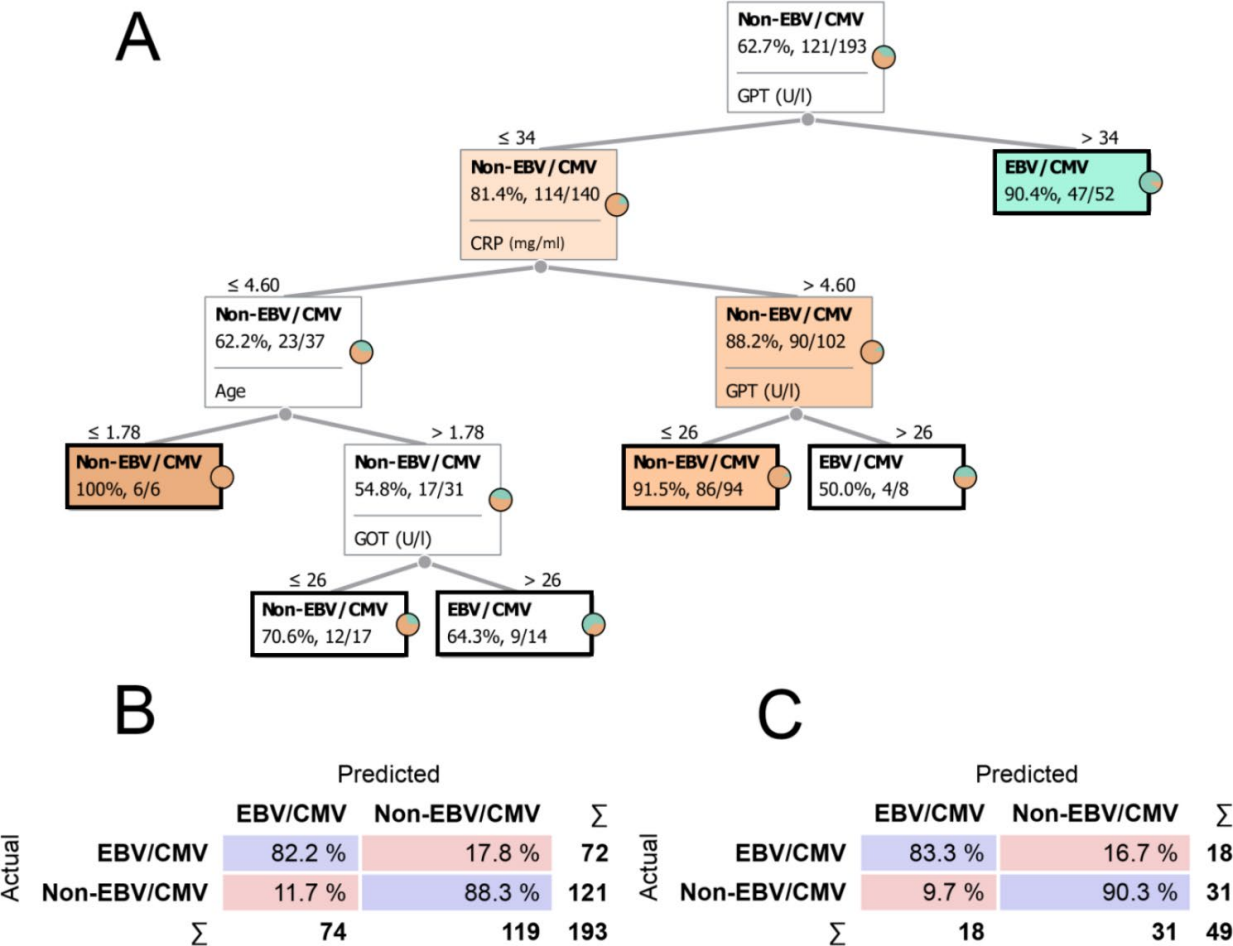
Classification tree based on symptoms. Part of the symptom-based classification tree (**A**): the nodes (rectangles) show the name and proportions of the most frequent group in each step. In the node at the top of the tree, the entire train set (n = 193) is still unpartitioned, in which non-EBV/CVM cases were the most common (62.70%, 121 of 192). At each step, patients are separated according to given symptoms to form more homogeneous nodes. A 0 denotes the absence of symptoms, while a 1 denotes their presence. Different intensities of the orange and green colours represent the percentage of non-EBV/CMV and EBV/CMV patients, respectively. Terminal nodes are marked with a black frames. Confusion matrices for test (**B**) and train set (**C**): each row of the matrices represents the instances in an actual class while each column represents the instances in a predicted class. Diagonally, the percentage of the correct classification is shown in blue. The percentage of errors is indicated in red. For example, a total of 72 EBV/CMV patients are shown in panel B, and of these 72, 82.2% were classified by the model into the EBV/CMV group and 17.8% into the Non-EBV/CMV group.242

To build the classification tree based on the parameters of the blood tests, the algorithm selected the GPT, CRP, GOT and age. The best results were obtained with 4 levels (Fig. 3A).

Running the model on the train dataset gave an average classification accuracy of 85.25% (Fig. 3B). The model classified 82.20% of EBV/CMV patients correctly and 17.80% incorrectly into the non-EBC/CMV group.

For the test set, the model correctly classified 83.30% of EBV/CMV patients, while it was wrong in 16.70%, resulting in an average classification accuracy of 86.80% (Fig. 3C). The positive and negative predictive value were measured as 83.33% and 90.32%, respectively.

According to the decision tree, 90.40% (47 of 52) of patients with a GPT level of 34 U/l belong to the EBV/CMV group.

The non-EBV/CMV group can be separated in several branches, but in the node with the highest number of patients, with CRP levels above 4.60 but GPT levels below 26.00, 91.5% of patients (86 of 94) belonged to non-EBC/CMV group.

In the third model, in which symptoms and laboratory results were considered together, the algorithm only considered blood test parameters as significant variables, and the same tree was obtained as in the model based solely on blood tests.

### The GPT could be a stand-alone marker for EBV/CMV infection

**Fig. 4:**
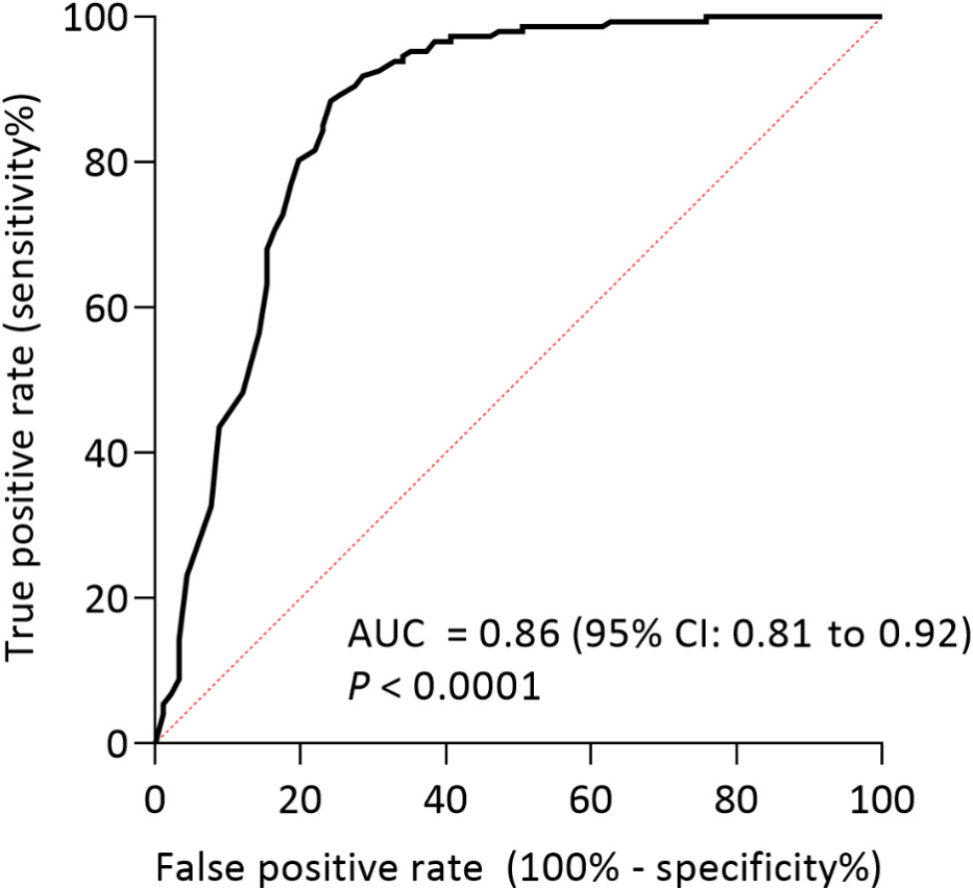
ROC analysis of the GTP. (AUC: area under the curve, CI: confidence interval, *P: p* value)

Based on the ROC analysis of the GPT, the AUC value was found to be 0.86 (95% CI: 0.81 to 0.91, *p* < 0.0001), the best cut-off point defined by the highest Youden index was 24.50 U/l, and the corresponding sensitivity and specificity were 88.44% and 75.82%, respectively (Fig. 4).

### Antibiotic treatments can be significantly reduced using our decision tree

In all patients with sore throat, viral infection was considered by physicians requesting EBV and/or CMV serology. In spite of this, antibiotics were still prescribed for 86.8% of patients in EBV/CMV group (79 of 91) before EBV or CMV infection was confirmed. In this group, amoxicillin, amoxicillin/clavulanic acid, penicillin-derivatives, cefuroxime, azithromycin, clarithromycin, and a combination of these antibiotics were most frequently prescribed (87.3%).

**Fig. 5:**
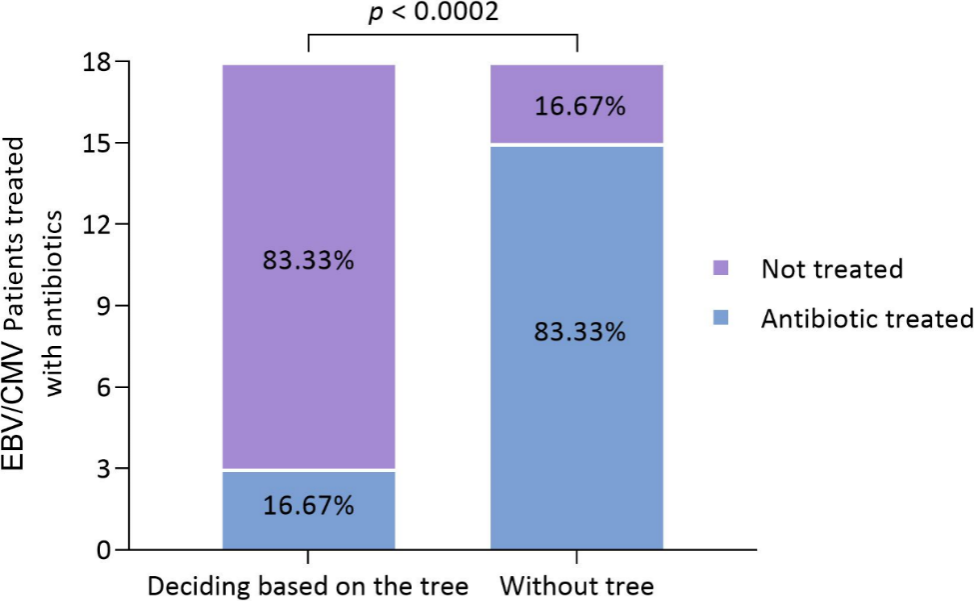
Proportion of the antibiotic treated patients in the test data set (n = 18). The figure shows the actual proportion of EBV/CMV patients in the test data set treated with antibiotics and the hypothetical proportion that would have been obtained using the classification tree. The *p* value is from Fisher’s exacttest

The test dataset used in classification included 36.73% EBV/CMV patients (18 of 49). 83.33% of EBV/CMV patients (15 of 18) received antibiotic treatment during clinical care. However, 13 EBV/CMV patients could have avoided antibiotic treatment if they had been diagnosed by the blood test-based classification tree constructed (Fig. 2A). This represents a 66.66% reduction in the number of EBV/CMV patients treated with antibiotics (*p* < 0.0002) (Fig. 5).

While there would be a significant reduction in antibiotic use in the EBC/CMV group, none of the patients with positive bacterial cultures would be deprived of antibiotic treatment.

## Discussion

Respiratory tract infections caused by a series of viral and bacterial agents are the second most common cause of morbidity in children and adults [7, 8]. Although viruses cause the majority of upper respiratory tract infections, antibiotics are used to treat a large proportion of patients. This is due in part to clinicians’ fear of bacterial over-infection, and in part to the difficulties in distinguishing viral from bacterial infections based on symptoms [3].

Antimicrobial resistance surveillance reports in Europe showed increasing resistance rates among gram-negative and gram-positive bacteria as well, and higher rates of resistance were reported in the south and east of Europe [9]. Increasing levels of resistance highlight the need for close monitoring and reducing the use of unnecessary antibiotic therapies.

Machine learning-based classification algorithms are increasingly used in clinical research to support diagnostic decision making. One such method is the classification tree, which offers a convenient way to implement flowchart-based classification. A number of published studies have demonstrated the effectiveness of classification trees in clinical research, mainly due to the fact that they have a solid theoretical background and are easy to interpret and apply [10–16].

In this study, we first examined the symptoms and blood test parameters of EBV- and/or CMV-infected patients compared to non-EBV/CMV group in the cohort studied, and then used the advantage of the decision tree to build a model that distinguishes EBV/CMV group from non-EBV/CMV patients. The aim of the study is to reduce unnecessary antibiotic use among children with tonsillopharyngitis.

In our study, serological tests showed that 37.60% of patients were infected with EBV or CMV (30.17% EBV, 2.48% CMV and 4.96% CMV or EBV). These findings are in line with the literature, which estimates the prevalence of EBV in children with tonsilitis from 9 to 58% [17, 18].

The cohort was also subjected to bacteriological culture. GAS positivity was identified in only 2.48% of the total cohort. This is somewhat lower than the prevalence of 15–30% reported in the literature, but is in line with studies that emphasise the overestimation of GAS in tonsillitis [17, 19–23].

One explanation for the observed low GAS prevalence could be the overuse of antibiotics, as physicians consider viruses as possible etiologic factors, but in most cases, they start antibiotic treatment as well, because of the longer turnaround time of serology, bacterial culture, and the fear of complications from streptococcal illness.

After forming the EBV/CMV and non-EBV/CMV groups based on the serological test, the different clinical parameters were investigated to assess which are characteristic for the EBV/CMV group.

Firstly, univariate methods were used to examine each symptom. The results showed that lymphadenitis, hepatosplenomegaly, exudation, sore throat are the more characteristic symptoms of the EBV/CMV group, and these frequencies proved to be significantly different compared to the non-EBV/CMV group.

Not surprisingly, as these symptoms are already well described and known features of EBV/CMV infections, and the prevalence we observed is consistent with the literature [24, 25]. In addition, we found that cough and nasal discharge were significantly less frequent in the EBV/CMV group compared to the non-EBV/CMV group. This finding is also consistent with the literature describing cough and runny nose as less common symptoms than lymphadenopathy, hepatosplenomegaly or exudation in children with EBV infection [26].

Analysing blood test parameters and age, we found that the mean levels of GOT and GPT were significantly higher in EBV/CMV patients, while the mean levels of CRP were lower on average (Fig. 1). This finding was confirmed by literature data that showed that over 80% of patients with EBV/CMV caused IM show elevated liver enzymes; thus, this can be used as a diagnostic marker for IM [27].

The elevated transaminase levels can be attributed to the liver damage caused by periportal lymphocyte infiltration and activated Kupfer-cells as part of a systemic reaction against viral infection [28].

Several studies have shown that CRP levels are higher in bacterial than viral upper respiratory tract infections [29]. However, our results cannot be fully interpreted in this light, as the non-EBV/CMV group does not only include cases of bacterial infection.

As with CRP, the significantly higher mean age in the EBV/CMV group cannot be compared with the published literature. It is well known that viral infections are significantly more common in older children compared to bacterial infections, but again, interpretation is difficult due to the heterogeneity of the non-EBV/CMV group [30].

After univariable statistical methods were used to explore the relationships between the different variables in the two groups, EBV/CMV and non-EBV/CMV, decision trees were constructed based on symptoms and blood test parameters.

Not surprisingly, the average classification performance of the blood test-based tree outperformed the symptom-based tree (Figs. 2 and 3).

It has already been pointed out that classification on the basis of symptoms is not sufficiently specific even when distinguishing viral infection from bacterial infection (Centor and FeverPAIN scoring) [6]. Our experimental design is difficult to compare with these findings, given that we did not aim to distinguish between viral and bacterial infections, but one type of viral infection from all others. However, this variance in symptoms may also explain why it is challenging to effectively differentiate tonsillopharyngitis caused by a certain virus from other pathogens.

In addition, hepatosplenomegaly was selected by the machine learning algorithm as one of the most important parameters of the symptom-based tree. However, this can only be established with the help of ultrasound, which makes the tree unsuitable for rapid decision-making support.

Apart from the lower efficiency of the symptom-based tree, according to the branches, the co-presence of hepatosplenomegaly, exudation, lymphadenopathy were found to be the most prominent and discriminative features for the EBV/CMV group (Fig. 2A). This result is consistent with those published in the literature and with univariable statistical analyses [24–26].

The blood test-based model, on the other hand, resulted in better performance. No difference was found between the classification efficiency of the train and test sets, suggesting that the differences observed in the train set between the EBV/CMV and non-EBV/CMV groups are generalizable and well applicable. These differences are mainly due to the levels of GPT and CRP. Based on the tree branches, GPT levels above 34 U/l are specifically characteristic of the EBV/CMV group. A high proportion of non-EBV/CMV patients showed a CRP level above 4.60 mg/ml and a GPT level below 26 U/l. Similar to the symptom-based tree, these results are also consistent with univariable statistical analyses and the available literature [27–30].

After GPT and CRP, another major branch starts based on age, after which GOT also plays a role in separation (Fig. 3A). However, this branching starting with age has a lower number of cases and is therefore may be less generalisable, and should be considered to be less emphasised than CRP and GPT in decision-making.

The question was raised, after GPT was found to be such an important variable in the decision tree, whether it could be an applicable marker of EBV/CMV infection. GPT could be a good performing stand-alone marker for the EBV/CMV group based on ROC analyses, as other studies have already pointed out (Fig. 4) [27]. However, it is not sufficiently specific: the specificity of the blood test-based tree was found to be 14.48% higher than GPT alone (90.3% vs. 75.82%). However, achieving adequate specificity (true negative rate) is crucial to reduce unnecessary antibiotic use.

Having found that the blood test-based decision tree has the best classification efficiency, we examined how much antibiotic use could be reduced in the test data set using the tree.

To do this, we compared the actual number of EBV/ECM patients receiving antibiotic treatment with the hypothetical number that would be achieved if the blood test-based tree had been used. The results showed that a significant reduction of 66.66% in unnecessary antibiotic use could be achieved.

In paediatric practice, the use of rapid antigen detection test (RADT) to prove the presence of GAS is recommended because of its ease of use, short turnaround time, and high specificity. However, because of its low sensitivity, bacteriological culture is used to confirm the diagnosis in cases of negative RADT. Because of the low sensitivity of RADT and long turnaround time of culture, clinicians face the dilemma whether to prescribe antibiotics or not [31].

In Hungary, the use of RADT to detect GAS in patients with pharyngitis is not common due to the lack of adequate financing, and physicians send throat swabs for culture if the laboratory is within easy distance. Therefore, the majority of GPs have no diagnostic test to distinguish between viral and bacterial pharyngitis, resulting in antibiotic prescription in over 80% of cases.

The use of the nucleic acid amplification test (NAAT) is currently not common in the diagnosis of acute pharyngitis; however, point of care (POC) NAATs may provide high sensitivity and specificity, and short turnaround time to establish a reliable diagnosis. The use of this method and RADT with culture can help physicians to decide on the need of antibiotic treatment [31]. However, because of the high sensitivity of NAATs, these tests can identify those patients in whom GAS is a coloniser rather than a true pathogen, thus this may lead to overuse of antibiotics, as well. In many countries, because of the relatively high cost of these molecular tests, the lack of specific training, and adequate financing, the use of NAATs at the POC by clinicians or their staff likely remains a big dilemma [32]-.

Our results have demonstrated that there is a significant discrepancy between confirmed diagnosis of bacterial tonsillopharyngitis and prescription of antibiotics. In order to reduce unnecessary antibiotic use, clear recommendations regarding the use of culture, serology, and new diagnostic methods are indispensable. Currently, in the absence of a single reliable and rapid differential diagnostic method, clinical and specific laboratory findings still play a crucial role in the distinction between viral and bacterial infections.

In this study, although we did not distinguish between viral and bacterial infections, we offer a blood test-based decision tree that can identify EBV/CMV infections with high specificity, thereby reducing unnecessary antibiotic treatment in children with tonsillopharyngitis.

Taking into account the obtained classification efficiency and the strong theoretical background of the applied algorithm, it would be worthwhile to improve the shortcomings of our study and to further develop the method so that it becomes suitable for distinguishing between bacterial and viral infections.

## Conclusion

Although identifying EBV/CMV through symptoms is difficult, a model with adequate specificity can be obtained using blood test parameters. Our study confirmed the importance of GTP, but our findings revealed that it has a higher specificity when several parameters are considered together. A decision tree built in this manner could help to reduce antibiotic use in children with tonsillopharyngitis.

## Electronic supplementary material

Below is the link to the electronic supplementary material.

Supplementary Material 1

Supplementary Material 2

Supplementary Material 3

## Data Availability

The datasets used and/or analysed during the current study are available from the corresponding author on reasonable request. The data are not publicly available due to privacy or ethical restrictions.
