# Supplementary material for: Diagnosis of Epstein-Barr and cytomegalovirus infections using decision trees: an effective way to avoid antibiotic overuse in paediatric tonsillopharyngitis
Source: BMC Pediatr. 2023 Jun 17;23:301. doi: 10.1186/s12887-023-04103-0 (PMC10276514; doi:10.1186/s12887-023-04103-0)
Supplement: Supplementary file 3 — Supplementary Material 3 [file 12887_2023_4103_MOESM3_ESM.docx]

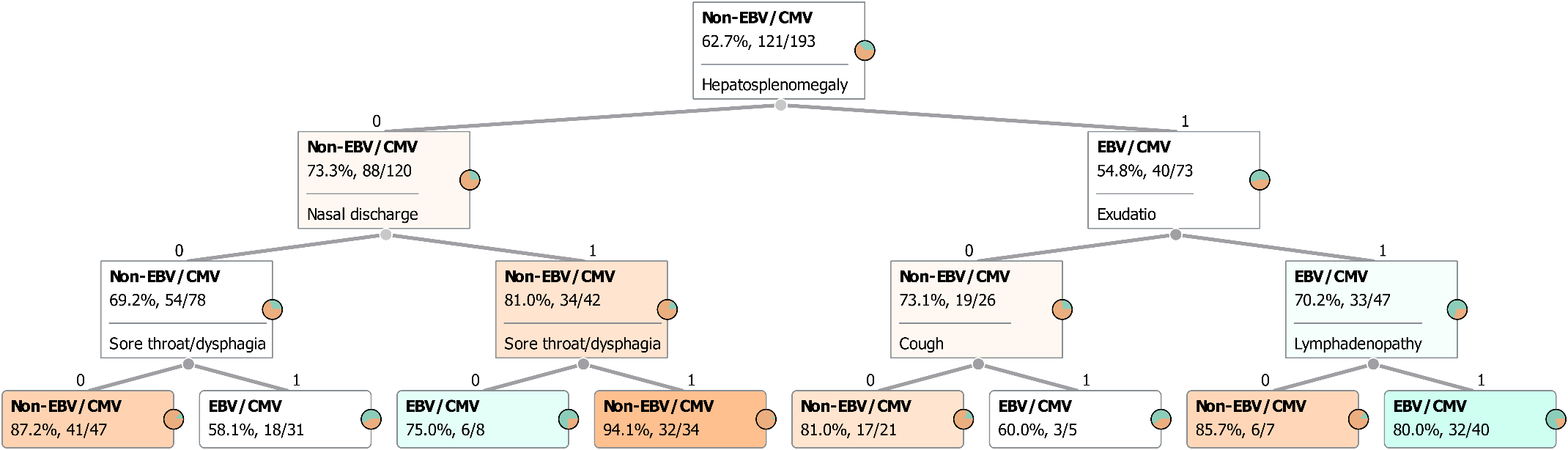


Additional file 3. Full version of the symptom-based classification tree. The nodes (rectangles) show the name and proportions of the most frequent group in each step. In the node at the top of the tree, the entire train set (n = 193) is still unpartitioned, in which non-EBV/CVM cases were the most common (62.70%, 121 of 192). At each step, patients are separated according to given symptoms to form more homogeneous nodes. A 0 denotes the absence of symptoms, while a 1 denotes their presence. Different intensities of the orange and green colours represent the percentage of non-EBV/CMV and EBV/CMV patients, respectively.
